# Terminal transfer amplification and sequencing for high-efficiency and low-bias copy number profiling of fragmented DNA samples

**DOI:** 10.1007/s13238-018-0540-9

**Published:** 2018-04-23

**Authors:** Dongqing Jiang, Xiannian Zhang, Yuhong Pang, Jianyun Zhang, Jianbin Wang, Yanyi Huang

**Affiliations:** 1 School of Life Sciences, Biodynamic Optical Imaging Center (BIOPIC), and Beijing Advanced Innovation Center for Genomics (ICG), Peking University, Beijing, 100871 China; 2 Department of Oral Pathology, School and Hospital of Stomatology, Peking University, Beijing, 100081 China; 3 School of Life Sciences, Tsinghua University, Beijing, 100084 China; 4 Center for Life Sciences, Beijing, 100871 China; 5 Materials Science and Engineering, College of Engineering, Peking University, Beijing, 100871 China

**Dear Editor,**

Since its invention, next generation sequencing (NGS) has greatly facilitated biomedical research and clinical diagnosis (Sikkema-Raddatz et al., 2013). Continuous dropping of the cost further accelerated the adaptation of sequencing as a standard analytical tool, from identification of drug candidates (Walker et al., 2015) to deciphering the complex biological systems (McConnell et al., 2013). However, progress in sample preparation technology has not been able to catch the speed of sequencing method evolution. For most NGS platforms, samples with DNA fragments to be sequenced need to be firstly converted into a ‘library’ in which each molecule can be further amplified into clones and then be sequenced. Library preparation is a critical step that generates short DNA fragments with certain adapters, and sometimes with barcodes, at both ends. While library construction from bulk genomic DNA samples is a routine procedure, traditional protocols become challenging when the starting material is limited. Materials from many research and diagnostic fields such as archaeology and preimplantation genetic diagnosis (PGD) (Treff et al., 2013) require DNA amplification before library construction.

Various related methods have been developed for whole genome amplification from minute amount of DNA, or even single cells. Methods involving random primers, such as MDA, DOP-PCR, or MALBAC, would get high yields yet still produce nonspecific amplification products (Marcy et al., 2007), incomplete coverage along genome and shortened DNA length (Fu et al., 2015). A recently development method, LIANTI (Chen et al., 2017), employed linear amplification and showed improved amplification evenness and less errors, compared to those exponential amplification protocols. However, all these methods require high quality starting material, and DNA from many types of samples such as chromatin immunoprecipitation (ChIP) products, formalin-fixed paraffin-embedded (FFPE) tissues, or ancient remains is highly fragmented. Short DNA could not be amplified efficiently by these methods.

Adapter-involving PCR strategy becomes more suitable for short-fragment samples by adding sequencing adapters directly to the ends of nucleotide strands through template-switching primers coupled with ligation. Various methods, such as LM-PCR (Dai et al., 2000), LinDA (Shankaranarayanan and Mendoza-Parra, 2011) and others (Liu et al., 2008) have been developed but they all facing the inevitable sample loss majorly because of incomplete ligation and repetitive purification. Hence handing small amount of starting DNA material is still challenging.

FFPE samples are always problematic for sequencing library preparation. Fixation, paraffin embedding, and archival storage conditions all contribute to fragmentation and other chemical damages of DNA. For FFPE samples, high efficient and short-length-specific library preparation methods are in great demand. In this paper, we present a versatile method for amplification and sequencing of minute amount of short-length DNA fragments. Our method, named Terminal Transfer Amplification and Sequencing (TTAS), relied on two rounds of tailing and amplification processes. TTAS is free of ligation step, independent of specific template DNA sequence, and compatible with prevalent sequencing library preparation protocols. We performed TTAS on fragmented genomic DNA from mouse embryo stem cells (mESCs) and HeLa cells and verified its amplification uniformity and efficiency. TTAS further demonstrated its capability of amplifying FFPE tissue-derived DNA from slices as small as 1 mm^2^.

For high throughput sequencing, it is always a challenge if the input material is short DNA since such short DNA is not suitable for prevalent protocols such as high efficient ligation or Tn5 tagmentation. We hence developed a novel method that employed a terminal elongation strategy to add universal tails at both ends of DNA for future PCR amplification (Fig. 1A). Since short DNA molecules generated by chemical degradation (eg., ancient DNA) or physical fragmentation (eg., sonication) may retain phosphate groups at 3′-terminus that prevented extension (Liu et al., 2008), template DNA requires dephosphorylation by phosphatase. We then used terminal deoxynucleotidyl transferase (TdT) to tail the 3′ end of template DNA with oligo deoxyadenosine (dA). A 20:1 mixture of dATP:ddGTP was used to control the tailing length. Multiple rounds of single oligo-T primer annealing and extension further amplify the tailed DNA in a linear fashion. Annealing was done at a relatively low temperature (48 °C) to facilitate the poly-A/oligo-T hybridization. The oligo-T primer contains an AcuI digestion site and a PCR priming sequence. A second round of terminal elongation further adds another poly-A tail to the end of each newly synthesized DNA molecule.

**Figure 1:**
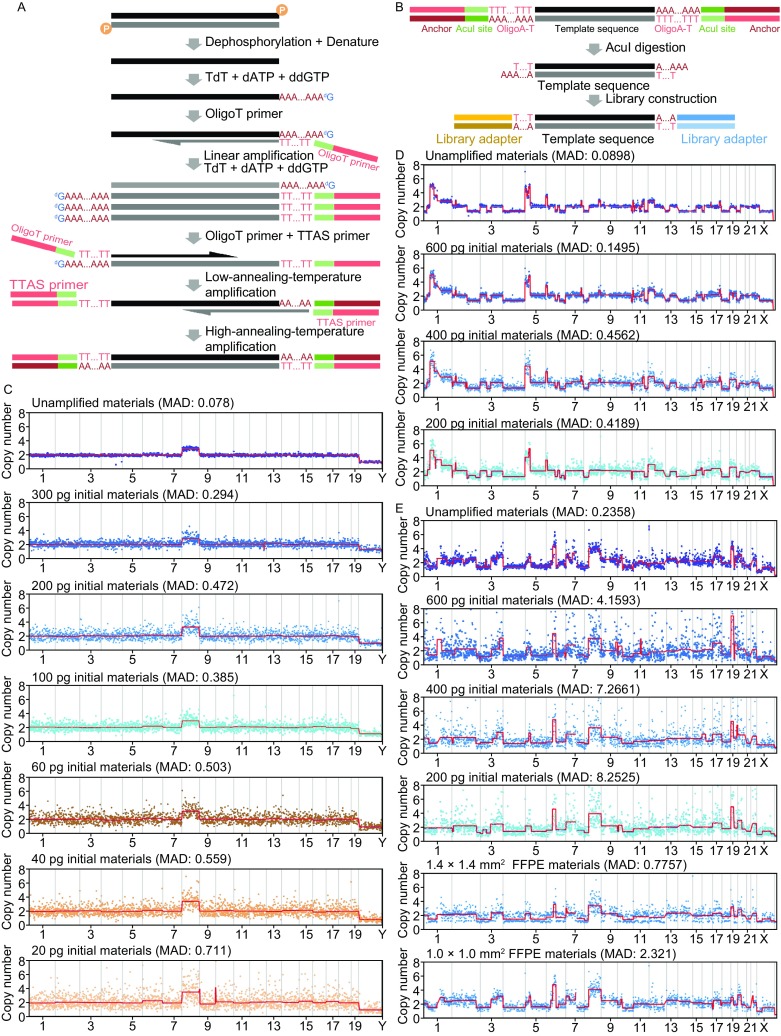
**Schematic illustration of TTAS protocol and copy number profiles of various samples**. (A) Protocol of DNA amplified by TTAS. (B) Schematic illustration of sequence structure of TTAS products. (C) Copy number profiles of mESC gDNA through TTAS, with different amount of starting material. Unamplified sample is used as control. (Plotted from 0.8 Gb sequence data. Bin size: 1 Mb) (D) Copy number profiles of HeLa gDNA samples through TTAS. (Plotted from 1 Gb sequence data. Bin size: 1 Mb) (E) Copy number profiles of FFPE samples through TTAS (Bin size: 1 Mb)

During amplification, oligo-T primers can also serve as templates and get tailed. It is therefore necessary to remove these primers before next round of PCR amplification. Purified ssDNA with 3′ poly-A tail was first subjected to several rounds of low-annealing-temperature linear amplification aiming to add anchor sequence to the oligo-A ends. Then we applied amplification based on anchor sequences at both ends, with higher annealing temperature, to boost the yield of the final product. Through amplification, the oligo-T-containing primers will generate two long homopolymeric A:T pairing sections in each amplicon. These homopolymeric regions will create difficulties during sequencing, not only sacrificing the informative read length but also lowering the sequencing quality. To remove such homopolymeric regions, we inserted two AcuI digestion sites that can be removed by the Acul restriction endonuclease together with 16 A-T basepairs. The resulting DNA products are suitable for standard Illumina library preparation (end repair and ligation) (Fig. 1B).

We first validated the amplification protocol using short DNA amplicons from PCR. 0.5 ng of 200-bp PCR product was subjected to the whole amplification process, and we noticed a size shift from ca. 200 bp to ca. 300 bp after two rounds of tailing and PCR amplification (Fig. S1A and S1B). Considering the sizing error, this measured shift agrees well with our design (92-bp size difference). We further sequenced the products by Sanger sequencing to confirm the amplification fidelity (Fig. S2).

Most highly fragmentized DNA samples are also limited in the quantity, making amplification and sequencing extremely challenging. It is therefore critical to strengthen the capture efficiency to minimize the material loss. In TTAS method, PCR anchor sequences are introduced by terminal elongation instead of ligation, which has been known to suffer from unsatisfied reaction efficiency (Cao et al., 2017). To minimize sample loss during purification, we optimized the experimental procedure and avoided unnecessary purification steps. TTAS protocol contains only one purification step before the main PCR cycles. These technical optimizations contribute to the high yield and good preservation of initial materials. In addition, TTAS is highly time-efficient, whereas most existing protocols are time consuming. In TTAS, the absence of ligation and bead binding (Liu et al., 2008) would substantially reduce the experimental time, making whole process accomplishable within 8 h.

For CNV profiling of limited amount of DNA, amplification has to be efficient with low bias. PCR can efficiently produce large amount of DNA copies. However, PCR also tends to associate with bias and errors, limiting its applications in many scenarios especially when the input sample is limited. In TTAS the linear amplification using oligo-T-containing primers has been proved to effectively reduce bias (Tang et al., 2009). Additionally, the primer sequences in the two rounds of linear amplification are identical, making the ends in amplification product complementary. Thus, DNA may form a stem-loop structure. This characteristic feature has been proved being able to effectively minimize bias during amplification (Picelli et al., 2013).

We next assessed the performance of TTAS on short genomic DNA fragments from cells. Genomic DNA was extracted from mESCs and fragmented by sonication (35 to 600 bp with peak around 210 bp) (Fig. S1C). We performed a titration experiment to better test the efficiency of TTAS on small amount of starting material. We achieved amplification of short DNA fragments with quantity as low as 20 pg and yielded more than 400 ng product for library construction. Length analysis showed that amplification products ranged between 100–800 bp with a peak at 300 bp (Fig. S1D). This length shift matched our expectation very well, indicating the successful amplification. The amplification products were converted to Illumina sequencing libraries for analysis. One of the noticeable features of TTAS is that it has low bias cross the whole genome, providing a suitable tool to unveil the copy number variations/alterations (CNVs/CNAs) of small amount of fragmented genomic DNA samples (Fig. 1C). With 1-Mb bin size, we can clearly see the faithful presentation of the chromosome copy numbers in the results from 20 pg starting DNA (20 pg sample of Fig. 1C).

To further explore TTAS’s capability of plotting complex CNV/CNA profiles and identifying smaller CNVs/CNAs from fragmented DNA samples, we further tested this protocol with sonication-fragmented HeLa genomic DNA (Fig. 1D). Amplification products from 200 pg DNA can retain major CNV/CNA events with 1-Mb bin size (200 pg sample of Fig. 1D).

TTAS is independent of the content or complexity of template sequences. DNA fragments are tailed nonspecifically by TdT to form poly-A tails for binding future primers. In the PCR step, the primers bind to the universal sequences that were introduced during two rounds of linear amplifications. This design guarantees uniform amplification across the whole genome and also endows TTAS a versatile method to amplify different types of DNA material. The tailing strategy also preserves the sequence information of the ends of DNA fragments. These regions are particularly desired for some short DNA sequencing applications, such as the identification of DNA damage hotspots and DNA replication stalling sites (Zhou et al., 2013).

DNA in FFPE samples are usually highly fragmented and contains various damages, and DNA extraction efficiency is typically limited. Such facts often cause difficulties for genomic analysis of these precious clinical samples, especially when the amount of starting material is limited. We applied TTAS on FFPE samples to evaluate its performance on difficult samples.

We chose a FFPE sample of human oral squamous cell carcinoma (OSCC) that was prepared 20 years ago. As expected, DNA extracted from the sample slice (1.5 cm by 1 cm by 10 μm) was highly fragmented (average size of a couple hundred base pairs) (Fig. S1G) and sequencing data revealed a distinct CNV profile (unamplified sample in Fig. 1E). Prevalent sequencing library preparation protocols for FFPE samples usually require more than 5 ng starting DNA. Such amount of DNA requires a large area, usually over 1 cm^2^, of 10 µm-thick slices. For those highly heterogeneous samples such as early stage cancers, such large-area slices will make the lesion-associated genomic variation obscured due to the low purity of cell types.

We first tested the lower limited of starting material for TTAS. Serial dilutions of bulk DNA from the FFPE samples underwent amplification and standard Illumina library construction. Sequencing data showed that 200 pg starting DNA was sufficient for TTAS to capture the major features of CNV profiles (200 pg sample in Fig. 1E). We next tested if TTAS can facilitate intratumor heterogeneity analysis by direct processing tiny sample sections. We sectioned the FFPE sample into 10 μm-thick slices and used laser capture microdissection (LCM) to capture specific tumor regions with different sizes. Each captured small piece of LCM sample typically contained merely 2,000 cells. To minimize the sequencing errors caused by DNA damage, we performed uracil-DNA glycosylase treatment (Do and Dobrovic, 2012) during DNA extraction from FFPE samples (Fig. S3). As shown in Figure 1E, TTAS retained the CNV profiles in sample section as small as 1 mm^2^ using 10 μm-thick slices. Our results showed that TTAS provided a unique solution to handle precious or limited samples, and to study intratumor heterogeneity through higher cellular purity ensured by LCM sampling.

The limitation of TTAS lies on the requirement of input material or, in the case of FFPE tissues, the size of the sample acquired by LCM. In our test, TTAS is able to generate high quality libraries from as little as 20 pg short-fragment DNA. Further reducing input amount of DNA has been difficult due to the nonspecific loss during purification after the limited-cycle linear amplification. For TTAS, a suggested smallest size of the FFPE LCM sample is 1 mm^2^.

We found that the fragment size of the input DNA also affected the performance of TTAS. During the second round of poly-A tailing, both amplified single strand DNA (ssDNA) and the residual free primers are tailed at 3′ end, making purification necessary to remove primers. If the fragment size of input material is too short (<100 bp), the size separation becomes difficult since the poly-A tailed primer will have a similar size as the true products. We found that FFPE samples, especially when treated with UDG, tend to have more primer dimers left after purification.

We also briefly tested if TTAS is suitable for special fragments from complex DNA samples, such as the fragmentized ChIP products. When applied TTAS with 300 pg of ChIP DNA from mouse tissues, we achieved 86.1% peak overlap between replicates, and with good concordance to reference. However, since ChIP products are special subsets of the whole genome, extensive optimization of TTAS is required for its future application on ultralow cell number ChIP products.

In summary, we have established a robust method, TTAS, to amplify and sequencing short DNA fragments with low bias and high efficiency. TTAS not only provides enough material for sequencing library construction, but faithfully retains the copy number profiles and terminal information of the DNA fragments. We optimized the TTAS protocol and test its efficiency with various fragmentized short DNA samples. Copy number profiling is achievable with 20 pg fragmented genomic DNA or 200 pg FFPE sample DNA. Direct processing of FFPE sample sections as small as 1 × 1 × 0.01 mm^3^ enables study of intratumor heterogeneity. We believe this method has a great potential for the amplification of various kinds of fragmentized DNA samples.

## Footnotes

We thank Dr. Xiannian Zhang and Dr. Yusi Fu for technical guidance and assistance, and Biodynamic Optical Imaging Center, Peking University High-throughput Sequencing Center for DNA sequencing assistance. We also thank Dr. Jie Shen for providing cells and Dr. Chen Cao for plant care. The genome sequence described and used in this research was derived from a HeLa cell line. Henrietta Lacks, and the HeLa cell line that was established from her tumor cells without her knowledge or consent in 1951, have made significant contributions to scientific progress and advances in human health. We are grateful to Henrietta Lacks, now deceased, and to her surviving family members for their contributions to biomedical research. This work was supported by National Natural Science Foundation of China (21327808, 21525521 to Yanyi Huang and 21675098 to Jianbin Wang), Ministry of Science and Technology of China (2015AA0200601 to Yuhong Pang and 2016YFC0900100 to Jianbin Wang and Yuhong Pang), and Beijing Advanced Innovation Center for Genomics.

Dongqing Jiang, Xiannian Zhang, Yuhong Pang, Jianyun Zhang, Jianbin Wang, and Yanyi Huang declare that they have no conflict of interest. All procedures followed were in accordance with the ethical standards of the responsible committee on human experimentation (institutional and national) and with the Helsinki Declaration of 1975, as revised in 2000 (5). Informed consent was obtained from all patients for being included in the study.

## Electronic supplementary material

Below is the link to the electronic supplementary material.

Supplementary material 1 (PDF 1987 kb)
