# Supplementary material for: Terminal transfer amplification and sequencing for high-efficiency and low-bias copy number profiling of fragmented DNA samples
Source: Protein Cell. 2018 Apr 23;10(3):229–33. doi: 10.1007/s13238-018-0540-9 (PMC6338616; doi:10.1007/s13238-018-0540-9)
Supplement: Supplementary file 1 — Supplementary material 1 (PDF 1987 kb) [file 13238_2018_540_MOESM1_ESM.pdf]

Supporting Information

Terminal transfer amplification and sequencing for high-efficiency and low-bias copy number profiling of fragmented DNA samples

Dongqing Jiang,[1] Xiannian Zhang,[1] Yuhong Pang,[1] Jianyun Zhang,[2] Jianbin Wang,[3,4,*] and Yanyi Huang[1,4,5,*]

[1]*School of Life Sciences, Biodynamic Optical Imaging Center (BIOPIC), and Beijing Advanced Innovation Center for Genomics (ICG), Peking University, Beijing, China.*

[2]*Department of Oral Pathology, School and Hospital of Stomatology, Peking University, Beijing, China*

[3]*School of Life Sciences, Tsinghua University, Beijing, China.*

[4]*Center for Life Sciences, Beijing, China.*

5Materials Science and Engineering, College of Engineering, Peking University, Beijing, China.

**Corresponding authors.*

E-mail: jianbinwang@mail.tsinghua.edu.cn (J.W.), or yanyi@pku.edu.cn (Y.H.)

Table of Contents

DNA Extraction from small sections of FFPE samples	2
Terminal transfer amplification procedure	2
Terminal-removal of amplification anchors by restriction endonuclease	3
Figure S1: Length comparison of unamplified and terminal transfer amplified DNA samples.	4
Figure S2: Sanger sequencing validation of terminal transfer amplified DNA from PCR amplicon	5
Figure S3: Schematic workflow for DNA extraction and TTAS from tiny sections of FFPE samples.	6

DNA Extraction from small sections of FFPE samples

Each region of interest from FFPE sample was collected into a 0.2 ml centrifuge tube containing 20 μl of deparaffinization solution (Qiagen) by Laser capture microdissection (LMD7000, Leica), followed by incubation at 56°C for 3 min. Then, the sample was washed twice by 50μl of 100% and 80% ethanol respectively for complete removal of paraffin. 1 μl of 10X Standard Taq buffer (NEB, B9014S), 2 μl of Protease K (PK, NEB, P8107S) and 7 μl of water (Ambion, 10977-015) was added as lysis buffer to the air-dried sample, and incubated at 56°C for 1 h, then 90°C for 1 h, 95°C for 10 min. Slightly centrifuged the tube before transferring all the solution into a new 0.2 ml centrifuge tube. 1μl of USER® Enzyme (NEB, M5505L) was added directly to the tube, mixed by pipetting and then incubated at 37°C for 1 h. All the mixture solution was used as initial DNA input to go through TTAS step.

Terminal transfer amplification procedure

The complete experimental procedure of terminal transfer amplification is following. To achieve a total volume of 10 μl, we concentrated the DNA solution to 8.5 μl and added 0.5 μl of 5 U/μl Antarctic Phosphatase (AnP, NEB, M0289S), 1 μl of 10X AnP buffer. The dephosphorylation reaction was carried out at 37˚C for 30 min then 70˚C for 5 min to inactivate AnP, followed by 95˚C for 1 min and transfered to ice immediately. PolyA was tailed by adding 0.3 μl of 15 U/μl Terminal Deoxynucleotidyl Transferase (rTdT, Invitrogen, 10533065), 2.7 μl of 5X TdT buffer, 0.3 μl of 10 μM dATP and 0.15 μl of 1μM ddGTP which was used to terminate tailing. The reaction was performed at 37˚C for 1 h and TdT was inactivated at 70 ˚C for 10 min.

Add 0.5 μl of 5 U/μl Antarctic Phosphatase, 0.5μl of 10X AnP buffer and 0.5 μl of water (Ambion, 10977-015) to the reaction mixture by flicking the tube with fingers, then incubate at 37˚C for 30 min to hydrolyse dATP and ddGTP followed by heating at 70˚C for 5 min to stop the reaction. Linear amplification was carried out in a total volume of 18 μl with the addition of 1.8 μl of 10X Standard Taq buffer, 0.3 μl of 5 U/μl Taq DNA polymerase (NEB, M0273S), 0.2 μl of 10 μM OligoT primer and 0.5 μl of 2 mM dNTP and 0.2 μl of water to the dephosphorylated sample. Initial denaturation was performed at 95˚C for 30 s, then 30 thermal cycles was followed involving 95˚C for 30 s, 48˚C for 30 s and 68˚C for 1 min. The amplified single strand DNA was dephosphorylated by adding 0.5 μl of 10X AnP buffer, 0.5 μl of 5 U/μl AnP and 1 μl of water. Incubate tubes at 37˚C for 30 min and inactivate AnP at 70˚C for 5 min.

The product was A-tailed by adding 3 μl of 5X TdT buffer, 0.5 μl of 15 U/μl rTdT, 3 μl of 10 μM dATP, 1.5 μl of 1μM ddGTP and 0.5 μl of water and incubated at 37˚C for 1 h followed by inactivation of rTdT at 70˚C for 10 min. Oligo(A)-tailed ssDNA was purified by DNA Clean & Concentrator™-5 kit (Zymo, D4003) according to the manufacturer's instruction manual that 7 volumes of DNA binding buffer was applied. Elute DNA in 15 μl of water.

Next, 14 μl of purified DNA was used for amplification in 22 μl reaction. PCR mix involving 2.2 μl of 10X Standard Taq buffer, 0.3 μl of 5 U/μl Taq DNA polymerase, 0.5 μl of 10 μM OligoT primer, 2 μl of 10 μM TTAS primer, 1.5 μl of 10 mM dNTP and 1.5 μl of water was added to the purified DNA. The thermal program was initiated at 95˚C for 30 s, then followed by 10 cycles of low-annealing-temperature amplification (95˚C for 30 s, 48˚C for 30 s, and 68˚C for 1 min) and 15 cycles of high-anneal-temperature amplification (95˚C for 30 s, 60˚C for 30 s, and 68˚C for 1 min), and finally primer extension for 2 min at 68˚C.

Finally, the amplified product was purified by DNA Clean & Concentrator™-5 kit using 5 volumes of DNA binding buffer and DNA was eluted with 20 μl water.

Terminal-removal of amplification anchors by restriction endonuclease

OligoT nucleotides of terminal transfer amplification product were removed by specific restriction endinuclease Acu I (NEB, R0641S) at CTGAAG site list in the table below for further application of library construction.

Primer Name	Primer Sequence (CTGAAG as AcuI site)
**OligoT Primer**	TAGGTTACGCAACGTTAACCACGTTAGCTGAAGTTTTTTTTTTTTTT
**TTAS Primer**	TAGGTTACGCAACGTTAACCACGTTAGCTGAAG

Sequencing libraries were constructed with NEBNext® Ultra DNA Library Kit for Illumina (NEB, E7370S), and sequenced on the Illumina HiSeq2500 platform following the manufacturers' instructions.

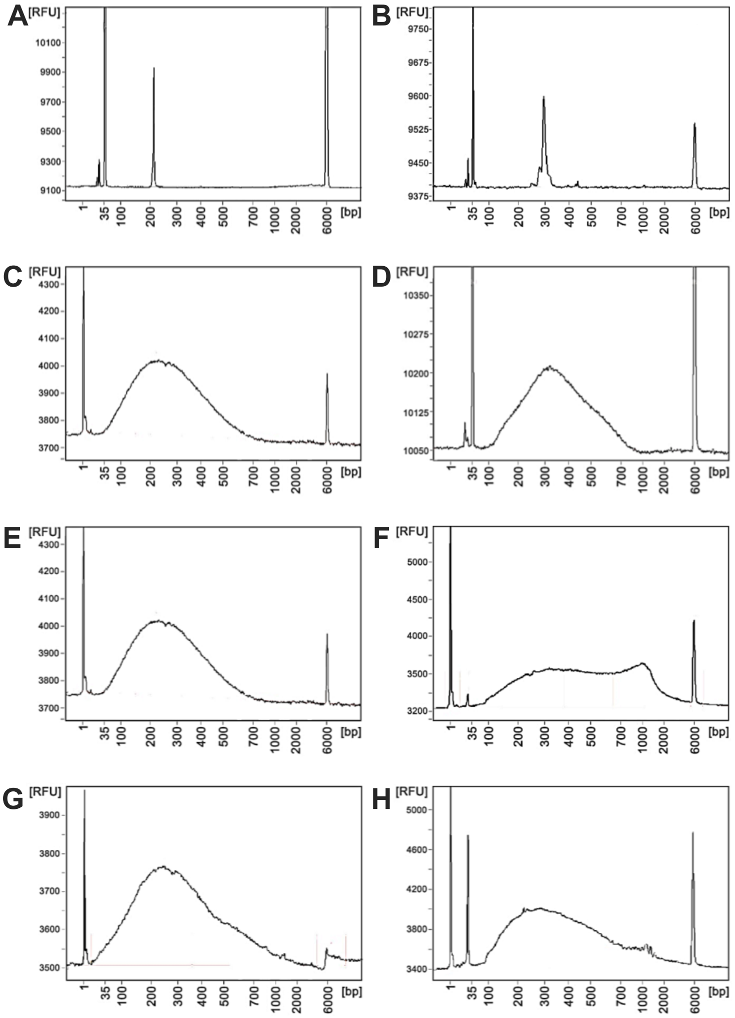

Figure S1: Length comparison of unamplified and terminal transfer amplified DNA samples. Fragment sizes are measured by capillary electrophoresis (Fragment Analyzer™ Automated CE System). (A, B) The length peak of short DNA amplicons from PCR was shifted from ca. 200 bp to ca. 300 bp after amplification. (C, D) Sheared mESC gDNA has a length distribution between 35 bp and 600 bp with a peak around 210 bp. After amplification the peak shifted to about 300 bp. (E, F) Amplification products of HeLa gDNA shows a longer size distribution than the unamplified gDNA. (G, H) Length measurement of unamplified and terminal transfer amplified DNA extracted from 1.5 cm by 1 cm by 10 µm FFPE samples. Amplified one conserved distribution shape of initial material with limited shift caused by terminal transfer amplification. (Marker peaks at 1 bp, 35 bp and 6000 bp represented lower and upper markers in DMS according to different kits.)

```
CGATTTGGTCAATGTGGAGGCGGCCACATGAGGAATT||TAGGTTACGCAAGGTTAACCACG
-------------------------------------||-----------------------

TTAGCTGAAGTTTTTTTTTTTTTTTTTTTAATAAACCAGCCAGCCGGAAGGGCCGAGCGC
-----------------------------AATAAACCAGCCAGCCGGAAGGGCCGAGCGC
                             *******************************

AGAAGTGGTCCTGCAACTTTATCCGCCTCCATCCAGTCTATTAATTGTTGCCGGGAAGCT
AGAAGTGGTCCTGCAACTTTATCCGCCTCCATCCAGTCTATTAATTGTTGCCGGGAAGCT
************************************************************

AGAGTAAGTAGTTCGCCAGTTAATAGTTTGCGCAACGTTGTTGCCATTGCTACAGGCATC
AGAGTAAGTAGTTCGCCAGTTAATAGTTTGCGCAACGTTGTTGCCATTGCTACAGGCATC
************************************************************

GTGGTGTCACGCTCGTCGTTTGGTATGGCTTCATTCAGCTCCGGTTCCAAAAAAAAAAAA
GTGGTGTCACGCTCGTCGTTTGGTATGGCTTCATTCAGCTCCGGTTCCC-----------
************************************************

AAAACTTCAGCTAACGTGGTTAACGTTGCGTAACCTA||A|ATCCCGCGGCCATGGCGGCCGG
-------------------------------------||----------------------

GAGCATGCGACGTCGGGCCCAAT
-----------------------
```

Figure S2: **Sanger sequencing validation of terminal transfer amplified DNA from PCR amplicon.** Upper line presents the Sanger sequencing results of terminal transfer amplified DNA, lower line presents the initial template sequence we designed. Star symbols (*) indicate perfect alignment, dash (-) indicate mismatch.

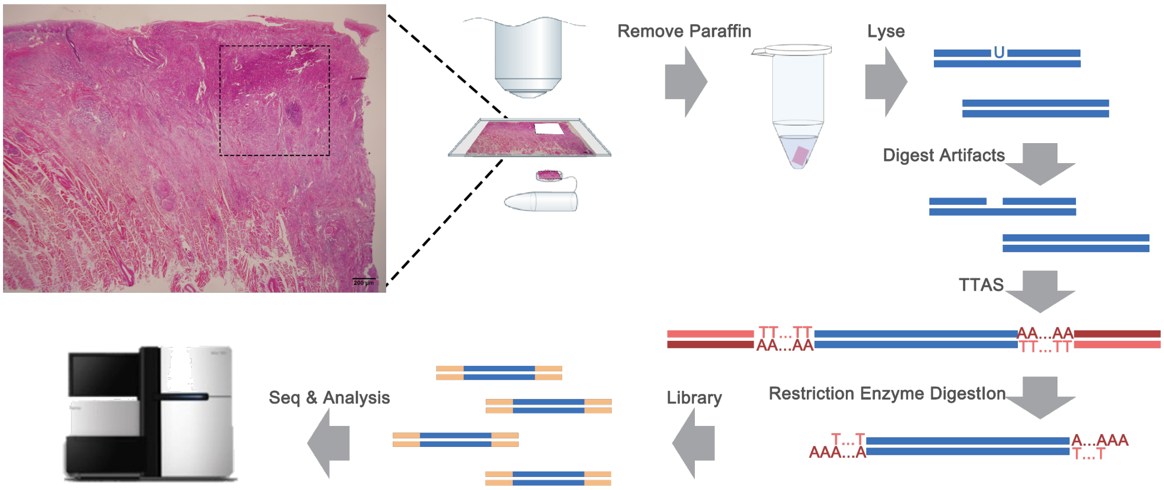

Figure S3: **Schematic workflow for DNA extraction and TTAS from tiny sections of FFPE samples.** Regions of interest were cut off by laser capture microdissection, followed by removal of paraffin, proteinase K digestion, and removal of artifact by USER® Enzyme. Then the content was used as initial DNA materials for subsequent TTAS.
